# High Performance Palladium Supported on Nanoporous Carbon under Anhydrous Condition

**DOI:** 10.1038/srep36521

**Published:** 2016-11-04

**Authors:** Zehui Yang, Ying Ling, Yunfeng Zhang, Guodong Xu

**Affiliations:** 1 Sustainable Energy Laboratory, Faculty of Materials Science and Chemistry, China University of Geosciences Wuhan, 388 Lumo RD, Wuhan, 430074, China

## Abstract

Due to the high cost of polymer electrolyte fuel cells (PEFCs), replacing platinum (Pt) with some inexpensive metal was carried out. Here, we deposited palladium nanoparticles (Pd-NPs) on nanoporous carbon (NC) after wrapping by poly[2,2′-(2,6-pyridine)-5,5′-bibenzimidazole] (PyPBI) doped with phosphoric acid (PA) and the Pd-NPs size was successfully controlled by varying the weight ratio between Pd precursor and carbon support doped with PA. The membrane electrode assembly (MEA) fabricated from the optimized electrocatalyst with 0.05 mg_Pd_ cm^−2^ for both anode and cathode sides showed a power density of 76 mW cm^−2^ under 120 °C without any humidification, which was comparable to the commercial CB/Pt, 89 mW cm^−2^ with 0.45 mg_Pt_ cm^−2^ loaded in both anode and cathode. Meanwhile, the power density of hybrid MEA with 0.45 mg_Pt_ cm^−2^ in cathode and 0.05 mg_Pd_ cm^−2^ in anode reached 188 mW cm^−2^. The high performance of the Pt-free electrocatalyst was attributed to the porous structure enhancing the gas diffusion and the PyPBI-PA facilitating the proton conductivity in catalyst layer. Meanwhile, the durability of Pd electrocatalyst was enhanced by coating with acidic polymer. The newly fabricated Pt-free electrocatalyst is extremely promising for reducing the cost in the high-temperature PEFCs.

Polymer electrolyte fuel cells (PEFCs) are sustainable future energy sources for portable and stationary applications due to the high-energy conversion efficiency and environmental friendliness^1^,^2^. However, the global commercialization of the PEFCs is still hindered by the high cost due to the use of platinum (Pt) as anode and cathode electrocatalysts, which is recognized as the most efficient catalyst for hydrogen oxidation reaction (HOR) and oxygen reduction reaction (ORR) and occupied 30% cost of the PEFCs^3^. Finding non-noble metal to replace Pt is one of the main issues in the PEFCs. Fortunately, palladium (Pd) is an attractive alternative due to the lower cost and similar properties compared to Pt (same group in periodic table, same fcc crystal structure, similar atomic size). More importantly, Pd is one of the most active catalysts for both HOR and ORR with the exception of Pt^4^,^5^,^6^,^7^,^8^. Thus it can be a good substitute for Pt as catalyst in fuel cells. It should be noted that Pd showed better ORR activity in alkaline medium and used as electrocatalyst in alkaline fuel cells (AFCs)^9^.

Numerous promising works have been published using Pd electrocatalyst in the direct methanol fuel cells (DMFCs), ORR test and direct formic acid fuel cells (DFAFCs). Mathiram *et al.* deposited Pd-NPs on carbon black (CB) and found the Pd/C showed superior fuel cell performance under high concentrated methanol compared to commercial CB/Pt since the Pd/C showed higher ORR activity under methanol penetrated from anode side^10^. Hoshi *et al.* systematically studied the activity of ORR on single crystal of Pd and pointed out that Pd(111) and Pd(100) showed comparable ORR activity to Pt(110) in acidic medium, indicating the Pd was able to potentially replace the Pt using in PEFC^11^. Also Pd has been proved that the activity for formic acid oxidation (FOR) was much higher compared to the Pt electrode because Pd directly oxidized formic acid to CO_2_, while, in the case of Pt, CO is generated as by-product and poisoned Pt^12^,^13^,^14^. While, no study has been reported about the Pd used in high temperature PEFC (HT-PEFC) which is the next-generation PEFC using phosphoric acid doped polybenzimidazole (PA-PBI) as electrolyte since the Nafion works under 80 °C with humidification^15^,^16^,^17^. HT-PEFC is promising since the high temperature benefits for the anti-CO poisoning of electrocatalyst, electrochemical reaction and water management^18^,^19^,^20^. Thus, in this work, we focused on the synthesis of new Pd-based electrocatalyst and used in the HT-PEFC.

Here, we selected nanoporous carbon (NC) as catalyst support due to the higher specific surface area facilitating the gas diffusion resulting in enhancement in fuel cell performance^21^. The electrocatalyst was synthesized *via* a bottom-up method, in which the NC was firstly wrapped by poly[2,2′-(2,6-pyridine)-5,5′-bibenzimidazole] (PyPBI), then doped with PA in order to form a homogeneous ionomer in the electrocatalyst and finally deposited Pd-NPs on polymer layer similar to the mechanism of Pt on PyPBI (Pt-N bonding) as shown in Fig. 1 22,23. The Pd size was controlled by varying the weight ratio between carbon support and Pd precursor and the fuel cell performance of the as-synthesized Pd electrocatalysts were evaluated under 120 °C without any humidification. To the best of our knowledge, this is the first study of Pd used in high-temperature PEFC. Finally the lower durability of the Pd electrocatalyst was enhanced by poly(vinylphosphonic acid) coating reported previously^21^,^24^,^25^,^26^.

## Results and Discussion

**Figure 2:**
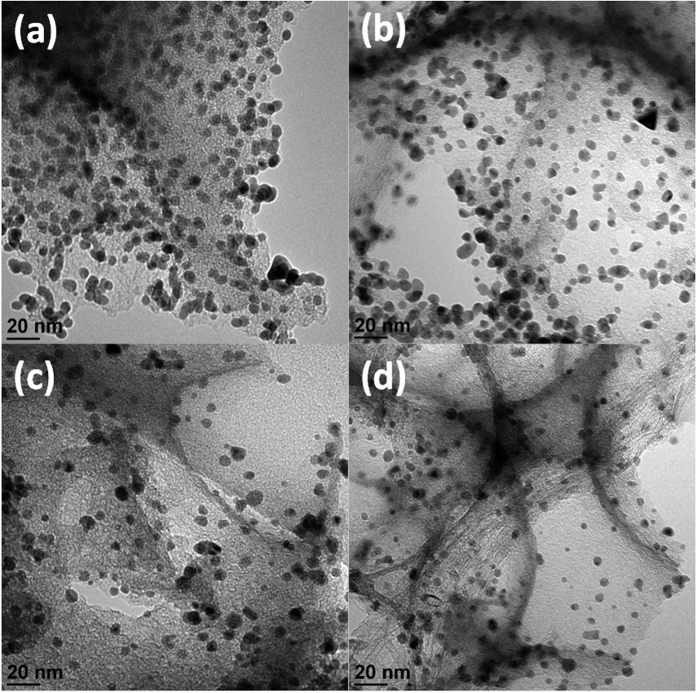
TEM images of newly synthesized electrocatalysts. TEM images of NC/PyPBI-PA/Pd_1_ (**a**), NC/PyPBI-PA/Pd_0.5_ (**b**), NC/PyPBI-PA/Pd_0.2_ (**c**) and NC/PyPBI-PA/Pd_0.1_ (**d**), respectively.

**Figure 3:**
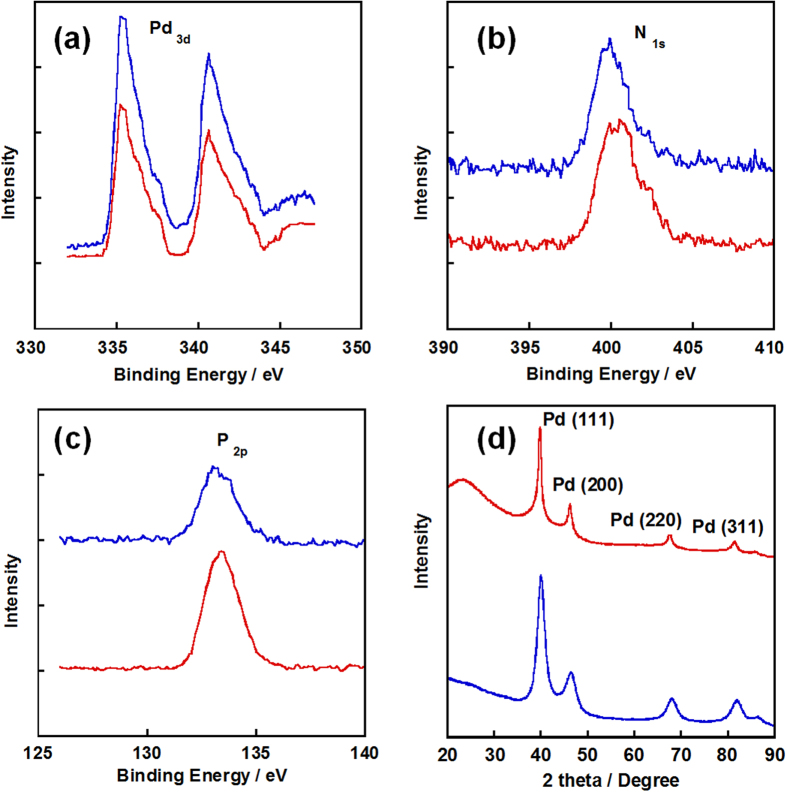
XPS spectra of newly synthesized Pd-electrocatalysts. (**a**) XPS narrow scan of NC/PyPBI-PA/Pd_1_ (blue line) and NC/PyPBI-PA/Pd_0.1_ (red line) in Pd_3d_ (**a**), N_1s_ (**b**) and P_2p_ (**c**) regions, respectively. (**d**) XRD patterns of NC/PyPBI-PA/Pd_1_ (blue line) and NC/PyPBI-PA/Pd_0.1_ (red line) measured from 20° to 90° with scan speed of 1° min^−1^.

Before the deposition of Pd-NP, the specific surface areas of NC and NC/PyPBI were measured and shown in Fig. S1, which were 1037 and 803 m^2^ g^−1^, respectively. The decrease in surface area indicated that the NC was successfully coated by PyPBI. As shown in Fig. 2, the Pd-NPs were homogeneous deposited on the PA doped NC/PyPBI (NC/PyPBI-PA) by the reduction of EG.aq at room temperature^27^. Also, the Pd-NPs diameter became smaller when the weight ratio between Pd precursor and NC/PyPBI-PA decreased since all the PyPBI covered on NC has the ability to anchor the Pd-NP by Pd-N bonding similar to Pt case reported previously^22^,^23^. The coordination between benzimidazole and Pd ion is of importance for Pd loading on the PyPBI^28^. And the strong interaction between Pd and PyPBI makes Pd stably anchor on NC. The diameters of Pd-NPs on NC/PyPBI-PA/Pd_1_, NC/PyPBI-PA/Pd_0.5_, NC/PyPBI-PA/Pd_0.2_ and NC/PyPBI-PA/Pd_0.1_ were 5.5±0.7 nm, 4.9±0.8 nm, 4.2±0.7 nm and 3.5±0.7 nm, respectively (for histogram see Supporting Information, Fig. S2). The interparticle distance became larger with the decreasing in weight ratio between Pd precursor and NC/PyPBI-PA due to the separation of Pd precursor in polymeric mediator as well as the weight ratio of Pd precursor to the carbon support since the PyPBI was homogeneously coated on carbon support and all the PyPBI is able to anchor the Pt nanoparticle. Similar phenomenon was observed in Pt case^26^. The enlarged interparticle distance is of importance for the oxygen reduction reaction (ORR) because enough space is needed for oxygen during the adsorption and reduction processes^29^,^30^. NC/PyPBI-PA/Pd_1_ and NC/PyPBI-PA/Pd_0.1_ have been selected for further measurements. The XPS measurements were carried out to determine the component in the electrocatalysts. The C_1s_ peak was calibrated at 284.5 eV mainly coming from carbon supports as shown in Fig. S3. As shown in Fig. 3a, two characteristic peaks observed at 335.5 and 341.0 eV in two electrocatalysts were assigned to 3d_5/2_ and 3d_3/2_ of Pd^0^, suggesting the reduction of Pd (II)^28^. And N_1s_ peaks were detected at 400 eV coming from the PyPBI shown in Fig. 3b^25^,^31^,^32^,^33^,^34^. Not surprisingly, the P_2p_ peaks were found at 132 eV shown in Fig. 3c due to the successful doping of NC/PyPBI with PA *via* acid-base interaction between PA and –NH on PyPBI^24^,^26^,^35^. Four diffraction peaks observed at 40°, 45.5°, 68° and 82° were assigned to Pd(111), Pd(200), Pd(220), and Pd(311), respectively as shown in Fig. 3d. The sizes of the crystal Pd were evaluated from XRD patterns based on the Scherrer equation (D = Kλ/(βcosθ)), where K is the shape factor, λ is the X-ray wavelength, β is the diffraction peak at half the maximum intensity, and θ is the diffraction angle), which was 4.5 nm and 3.1 nm for NC/PyPBI-PA/Pd_1_ and NC/PyPBI/Pd_0.1_, respectively, suggesting the successfully reduction of Pd(II).

**Figure 4:**
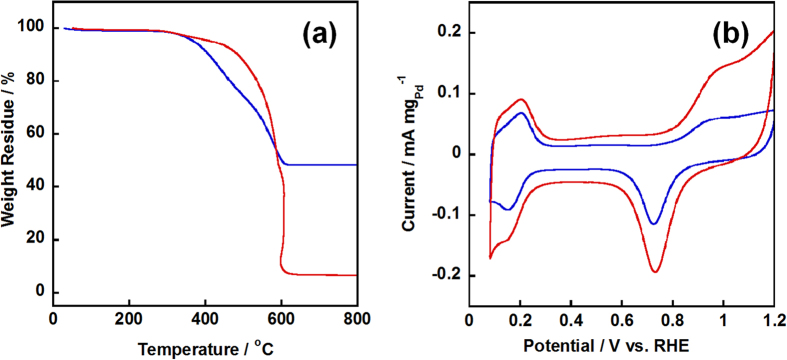
TGA curves and electrochemical measurements of Pd-electrocatalysts. (**a**) TGA curves of NC/PyPBI-PA/Pd_1_ (blue line) and NC/PyPBI-PA/Pd_0.1_ (red line) measured under stable air-flow (100 mL min^−1^) with heating rate of 10 °C min^−1^. (**b**) CV curves of NC/PyPBI-PA/Pd_1_ (blue line) and NC/PyPBI-PA/Pd_0.1_ (red line) measured in N_2_-saturated 0.1M HClO_4_ electrolyte.

Before the electrochemical analysis, the Pd amounts were confirmed by the TGA measurement, which was carried out under air flow to determine the Pd loading amount in the composite. As shown in Fig. 4a, the Pd loading amounts were 48.1 wt% and 5.5 wt% for NC/PyPBI-PA/Pd_1_ and NC/PyPBI-PA/Pd_0.1_, respectively. Also the Pd amounts evaluated from the inductively coupled plasma emission spectrometer (ICP-AES, Shimadzu) were 47.3 wt% and 4.9 wt% for NC/PyPBI-PA/Pd_1_ and NC/PyPBI-PA/Pd_0.1_, respectively, which were comparable to the values measured by TGA. Based on the feeding of Pd precursor, the theoretical loading amounts were 47.5 wt% and 4.8 wt% for NC/PyPBI-PA/Pd_1_ and NC/PyPBI-PA/Pd_0.1_, respectively, indicating almost all the Pd were successfully loaded on the NC/PyPBI-PA at room temperature. Cyclic voltammetry was carried out by potential cycling from 0.1 V to 1.2 V *vs*. RHE in N_2_-saturated 0.1M HClO_4_ electrolyte. In order to make a fair comparison, the electrocatalyst loading on glassy carbon electrode (GCE) was same in order to form same thickness of the catalyst film. As shown in Fig. 4b, one peak couple at 0.1 V to 0.3 V *vs*. RHE was attributed to the electro-adsorption and -desorption of hydrogen on Pd metal and the other peak couple at 0.7 V to 1.2 V *vs*. RHE came from the oxidation and reduction of Pd metal. The larger hydrogen electro-adsorption peak obtained from NC/PyPBI-PA/Pd_0.1_ was due to the smaller Pd-NPs size, suggesting the smaller Pd-NPs enhanced the hydrogen oxidation reaction (HOR). The most common and convenient way to determinate the electrochemical surface area (ECSA) of Pd electrocatalyst involves the charge of formation and reduction PdO based on equation: ECSA = Q_H_/(424×Pd loading amount), the charge density of 424 μC cm^−2^ is associated to the reduction of the formed PdO monolayer^36^. Thus, the ECSAs of NC/PyPBI-PA/Pd_1_ and NC/PyPBI-PA/Pd_0.1_ were 57.7 and 115.8 m^2^ g_Pd_^−1^, respectively. Due to the smaller Pd size of NC/PyPBI-PA/Pd_0.1_, the ECSA was almost twice higher compared to that of NC/PyPBI-PA/Pd_1_. As well known, the Pd utilization efficiency is calculated from equation: η = 6/(ρd*ECSA)^37^, where ρ and d are the density and diameter of Pd, respectively. Thus, the Pd utilization efficiency of NC/PyPBI-PA/Pd_0.1_ was reached 81.1%, which was much higher that of NC/PyPBI-PA/Pd_1_ (63.5%). The Pd utilization efficiency was improved using this method which was similar to Pt cased reported previously by us^26^.

**Figure 5:**
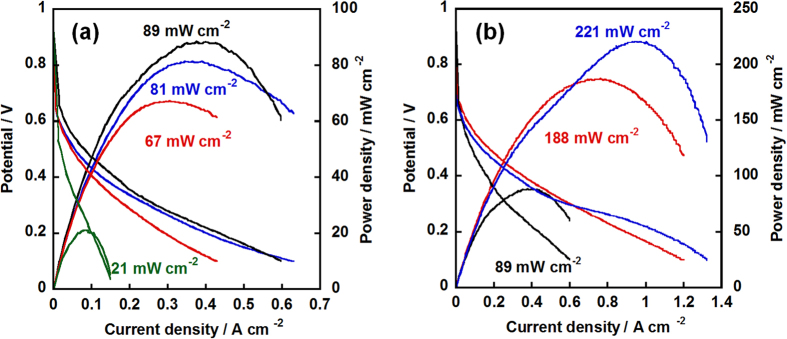
Fuel cell performances of Pd electrocatalysts and commercial CB/Pt. (**a**) I-V and power density curves of the MEAs fabricated from commercial CB/Pt (anode and cathode, 0.45 mg_Pt_ cm^−2^, black line), NC/PyPBI-PA/Pd_1_ (anode and cathode, 0.45 mg_Pd_ cm^−2^, blue line), NC/PyPBI-PA/Pd_0.1_ (anode and cathode, 0.05 mg_Pd_ cm^−2^, red line) and commercial CB/Pt (anode and cathode, 0.05 mg_Pt_ cm^−2^, green line), respectively. (**b**) I-V and power density curves of the hybrid MEAs fabricated from commercial CB/Pt (cathode, 0.45 mg_Pt_ cm^−2^) as cathode and NC/PyPBI-PA/Pd_1_ (anode and cathode, 0.45 mg_Pd_ cm^−2^, blue line) and NC/PyPBI-PA/Pd_0.1_ (anode, 0.05 mg_Pd_ cm^−2^, red line) as anode, respectively. The maximum power densities were shown.

**Figure 6:**
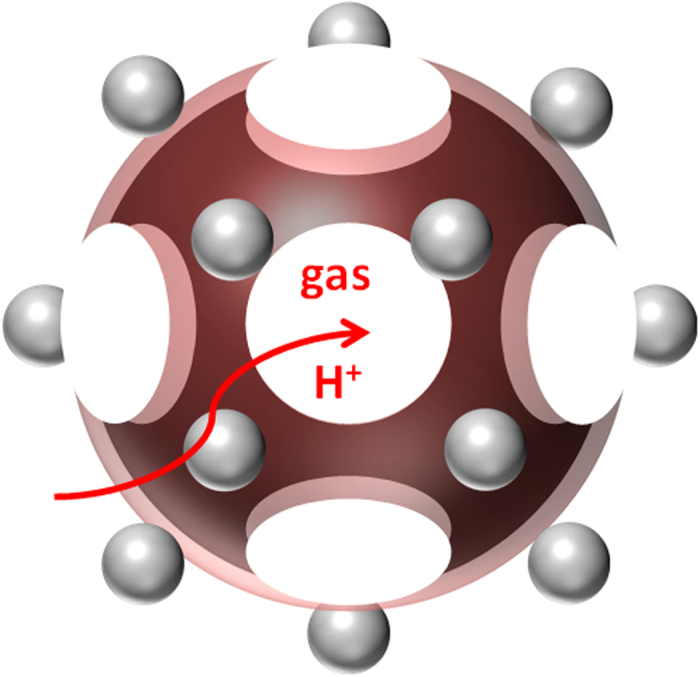
Schematic illumination of Pd-electrocatalyst. Schematic illumination of enhancement in fuel cell performance of NC/PyPBI-PA/Pd electrocatalyst.

The activity of the electrocatalyst was evaluated under 120 °C without any humidification by using membrane electrode assembly (MEA) sandwiched from PA-doped PBI electrolyte and two gas diffusion electrodes (GDEs) fabricated from newly synthesized electrocatalyst. The MEAs is tested under high temperature with anhydrous condition which is the target of the next-generation PEFC because high-temperature is favor for the electrochemical reaction, water management and anti-CO poisoning. In order to simulate the real practical operation, dry hydrogen and air were flowed to anode and cathode sides, respectively. To eliminate the thickness effect, the loading amounts of NC/PyPBI-PA/Pd_1_ and NC/PyPBI-PA/Pd_0.1_ were controlled to be 0.45 mg_Pd_ cm^−2^ and 0.05 mg_Pd_ cm^−2^, respectively, because the Pd amount in the electrocatalyst were different (48.1 wt% and 5.5 wt%). The MEA fabricated from commercial CB/Pt with 0.45 mg_Pt_ cm^−2^ loading was used as control sample. Interestingly, the fuel cell performance of NC/PyPBI-PA/Pd_0.1_ was 76 mW cm^−2^, which was comparable to those of NC/PyPBI-PA/Pd_1_ (81 mW cm^−2^) and commercial CB/Pt (89 mW cm^−2^) as shown in Fig. 5a. The resistances of the three MEAs were similar during the measurements as shown in Fig. S4a. Also the MEAs fabricated from the non-PA-doped electrocatalysts (for XPS survey scans, see Supporting Information, Fig. S5) were estimated as shown in Fig. S6. The maximum power densities of NC/PyPBI/Pd_1_ (0.45 mg_Pd_ cm^−2^ for anode and cathode) and NC/PyPBI/Pd_0.1_ (0.05 mg_Pd_ cm^−2^ for anode and cathode) were 46 and 18 mW cm^−2^, respectively. The PA-doped electrocatalysts showed higher power densities due to PA doping since the PyPBI-PA efficiently transfers the proton in the catalyst layer since non-doped PyPBI has very low proton conductivity^35^. Thus, the homogeneous proton conductor in the catalyst layer is essential for the improvement in fuel cell performance. The comparable power densities of Pd-based MEA to commercial CB/Pt were attributed to two main reasons, namely, i) porous structure and ii) PyPBI-PA in the catalyst layer, which benefited the gas diffusion and proton transfer in the catalyst layer resulting in higher utilization efficiency of the electrocatalyst as schematically shown in Fig. 6. In order to emphasize the higher activity of the as-fabricated electrocatalyst, the maximum power density of MEA fabricated from commercial CB/Pt with 0.05 mg_Pt_ cm^−2^ loading was measured (21 mW cm^−2^, Fig. 5a), which was triple times lower compared to the MEA fabricated from NC/PyPBI-PA/Pd_0.1_ with 0.05 mg_Pd_ cm^−2^. The higher power density attributed to the easier gas diffusion and proton transfer. The MEA fabricated from NC/PyPBI-PA/Pd_0.1_ (0.05 mg_Pd_ cm^−2^) showed comparable power density to that of the commercial CB/Pt (0.45 mg_Pt_ cm^−2^), suggesting that NC/PyPBI-PA/Pd_0.1_ is promising substitute for Pt electrocatalyst used in HT-PEFC.

As well known, Pt shows higher ORR activity than Pd in the acidic medium and fuel cell performance mainly depends on the ORR activity since hydrogen oxygen reaction (HOR) is 10^7^ times faster than ORR^38^. Thus, we switched the cathode electrocatalyst to commercial CB/Pt to prepare the hybrid MEAs. The power density of hybrid MEA fabricated from NC**/**PyPBI-PA/Pd_0.1_ with 0.45 mg_Pd_ cm^−2^ for anode and commercial CB/Pt (0.45 mg_Pt_ cm^−2^) for cathode reached 221 mW cm^−2^, which was somewhat higher compared to the MEA from NC/PyPBI-PA/Pd_0.1_ with 0.05 mg_Pd_ cm^−2^ for anode and commercial CB/Pt (0.45 mg_Pt_ cm^−2^) as shown in Fig. 5b with similar resistances as shown in Fig. S4b. The hybrid MEAs showed almost twice higher power density than Pd-based MEA due to higher ORR activity of commercial CB/Pt. Notably, the hybrid MEAs showed more than twice higher power density compared to commercial CB/Pt suggesting that the homogeneous proton transfer (PA-PyPBI) in catalyst layer is essential for the enhancement in fuel cell performance. The PA is inhomogeneous in the catalyst layer fabricated from the commercial CB/Pt because the commercial CB/Pt has no binding site for PA and the PA is mobile in the catalyst layer of commercial CB/Pt resulting in lower utilization of electrocatalyst. The hybrid and Pd-based MEAs are promising candidates for HT-PEFC since the dosage of Pt was highly decreased and the power density obtained at low Pt usage was higher compared to the recent published values tested in HT-PEFC as shown in Table 1.

**Figure 7:**
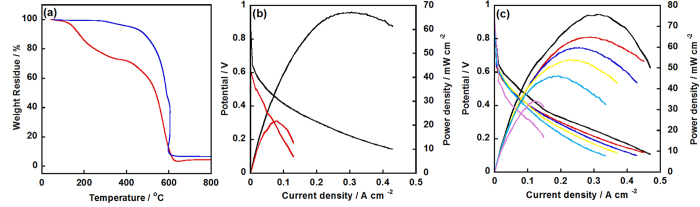
TGA curves of Pd-electrocatalyst before and after PVPA coating and durability results. (**a**) TGA curves of NC/PyPBI-PA/Pd_0.1_ (blue line) and NC/PyPBI-PA/Pd_0.1_/PVPA (red line), respectively. I-V and power density curves of NC/PyPBI-PA/Pd_0.1_ (**b**) and NC/PyPBI-PA/Pd_0.1_/PVPA (**c**) after 1 (black line), 5000 (red line), 10000 (blue line), 15000 (yellow line), 20000 (green line) and 25000 (pink line) potential cycles from 1.0 V to 1.5 V *vs*. RHE.

**Figure 8:**
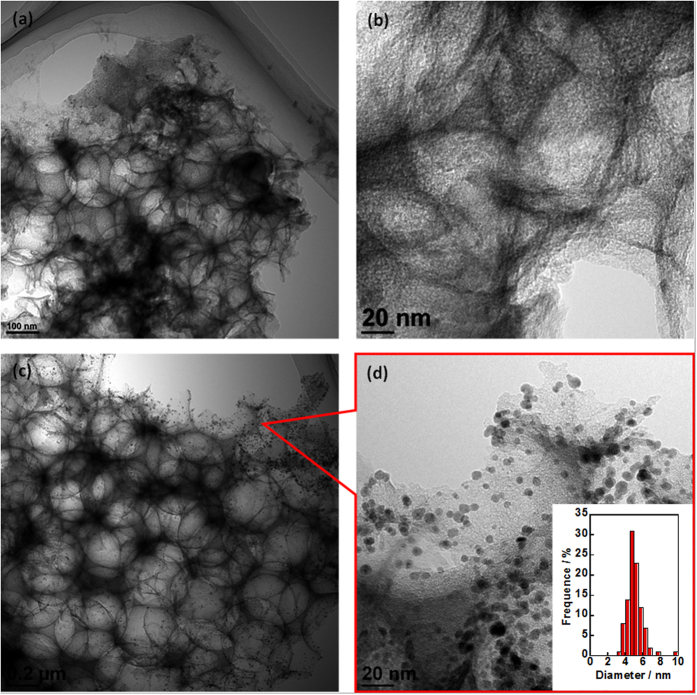
TEM images of PVPA coated and non-coated electrocatalyst after durability test. TEM images of NC/PyPBI-PA/Pd_0.1_ (**a**,**b**) and NC/PyPBI-PA/PyPBI/Pd_0.1_/PVPA (**c**,**d**) after durability test with low and high magnification.

It should be noted that the Pd-NP was not very stable under acidic medium, as reported previously, polymer^24^,^34^ coating would be efficient for the enhancement in long-term durability. Thus, the NC/PyPBI-PA/Pd_0.1_ was selected and coated with poly(vinylphosphonic acid) (PVPA) to form NC/PyPBI-PA/Pd_0.1_/PVPA. As shown in Fig. 7a, the Pd amount was decreased from 5.5 wt% to 4.3 wt% after coating with PVPA, which was evaluated to be 21.8 wt%. The PVPA coated electrocatalyst was used to fabricate the MEA and tested the durability of MEA by potential cycling from 1.0 V to 1.5 V *vs*. RHE, in which the carbon corrosion process is accelerated resulting in detachment of metal on carbon support. The power densities of two different MEAs at various temperatures were compared as shown in Fig. S7. The maximum power densities of NC/PyPBI-PA/Pd_0.1_/PVPA at 120 °C, 140 °C and 160 °C were 76, 96 and 129 mW cm^−2^, which were higher compared to those of NC/PyPBI-PA/Pd_0.1_ (60, 95, 109 mW cm^−2^ at 120 °C, 140 °C and 160 °C). The higher power densities of NC/PyPBI-PA/Pd_0.1_/PVPA compared to those of NC/PyPBI-PA/Pd_0.1_ were due to the PVPA layer, which is a conductive polymer under high temperature and the proton can be efficiently delivered by PVPA and PyPBI-PA. The durability test was performed under 120 °C as shown in Fig. 7. After 25,000 potential cycles, the maximum power density was decreased to 34 mW cm^−2^ with similar resistance during the durability test as shown in Fig. S8, which was 5 times higher compared to MEA from NC/PyPBI-PA/Pd_0.1_ (Fig. 7b) because the PVPA and PyPBI layer stabilized the Pd-NP similar to Pt-NP. In order to confirm the existence of Pd-NP after durability test, electrocatalyst from cathode was detected by *ex-situ* TEM. As shown in Fig. 8(a,b), Pd-NP was almost dissolved during the potential in the absence of PVPA, while, the Pd-NP was still observed and the diameter was grown to 5.0±0.8 nm (Fig. 8(c,d)). Thus, the PVPA was an efficient way to stabilize the newly synthesized Pd electrocatalyst. The Pd electrocatalyst was promising candidate electrocatalyst for application in HT-PEFC.

## Conclusions

In conclusion, we successfully deposited Pd-NPs on NC/PyPBI doped with PA and varied the Pd-NP size by changing the weight ratio between Pd precursor and carbon support. The MEA fabricated from Pd-based electrocatalyst (0.05 mg_Pd_ cm^−2^ loaded in anode and cathode) showed comparable power density to commercial CB/Pt (0.45 mg_Pt_ cm^−2^ loaded in anode and cathode) under 120 °C without any humidification. Moreover, the hybrid MEA prepared from NC/PyPBI-PA/Pd_0.1_ with 0.05 mg_Pd_ cm^−2^ loaded in anode and commercial CB/Pt with 0.45 mg_Pt_ cm^−2^ loaded in cathode showed a high power density of 188 mW cm^−2^. The porous structure and homogeneous PA doping assisted by PyPBI enhanced the gas diffusion and proton transfer leading to the high fuel cell performance. Also the dissolution of Pd electrocatalyst decelerated by PVPA coating was proved. The newly synthesized Pd electrocatalyst is promising for lowering the cost of HT-PEFC.

## Method

### Materials

Palladium acetate (Pd(OAc)_2_), 2-propanol, *N,N*-dimethylacetamide (DMAc), ethyl alcohol ethylene glycol (EG), Poly(vinylphosphonic acid) (PVPA, 30 wt%) and 85% phosphoric acid (PA) were purchased from Sinopharm Chemical Reagent Co., Ltd. Commercial CB/Pt (Pt amount: 37.9 wt%) was obtained from Tanaka Kikinzuku Kogyo KK. All the chemicals were used as received without any purification. NC was synthesized according to previous reports^39^. PyPBI and poly[2,2′-(2,6-phenyl)-5,5′-bibenzimidazole] (PBI) were synthesized according to previously reported methods^40^.

### Synthesis of electrocatalyst

10 mg of NC was dispersed in 20 mL DMAc by sonication for 1 h. 5 mg of PyPBI was dissolved in 10 mL DMAc by stirring for 2 h. These solutions were then mixed and sonicated for 2 h, then filtered and dried overnight under vacuum at 80 °C. The obtained composite (NC/PyPBI) was dispersed in water by sonication and 1 mL PA (85%) was added. The solution was stirred for 1 h at room temperature and collected by filtration and drying under vacuum at 80 °C. The deposition of Pd-NPs was carried out by the reduction of Pd(OAc)_2_ in EG aqueous solution (EG:H_2_O = 3/2,*v/v*). First, 13.5 mg of Pd(OAc)_2_ was dissolved in a 5 mL EG.aq and 10 mg of NC/PyPBI-PA dispersed in 50 mL EG.aq was added. The mixture was stirred at room temperature for 3 h under N_2_ atmosphere. By filtration of the dispersion, we obtained the electrocatalysts, which were dried overnight in oven at 80 °C to remove the remained solvent.

### Synthesis of PVPA-coated electrocatalyst

10 mg of the as-prepared electrocatalyst was dispersed in water (20 mL) by sonication for 10 min and 1 mL of PVPA solution was added. The mixture was sonicated for another 10 min. The PVPA-coated electrocatalyst was collected by filtration, washing by water and drying at 80 °C under vacuum for 8 h.

### Characterization

The X-ray photoelectron spectroscopy (XPS) spectra were measured using an AXIS-ULTRA^DLD^ (Shimadzu) instrument. The TGA measurements were conducted using an EXSTAR 6000, Seiko Inc., at the heating rate of 5 °C min^−1^ under 100 mL min^−1^ of air flow. The TEM images were measured using a JEM-2010 (JEOL, acceleration voltage of 120 kV) electron microscope. A copper grid with a carbon support was used for the TEM observations. X-ray diffraction (XRD) measurements were carried out by using a Rigaku SmartLab diffractometer (Cu, Kα, λ = 1.5406 Å, 40 kV, and 30 mA), the diffraction patterns were collected from 20° to 90° at a scan rate of 1° min^−1^ and with a step of 0.01°.

### Electrochemical measurements

The electrochemical measurements were performed using a glassy carbon electrode (GCE) attached to CHi604e instrument with a conventional three-electrode system in a vessel at 25 °C. A GCE with a geometric surface area of 0.196 cm^2^ was used as the working electrode. A Pt wire and an Ag/AgCl were used as the counter and reference electrodes, respectively. The potential of the electrode was controlled by a CHI604e potentiostat. The catalyst ink was typically prepared as follows. The prepared catalyst (1.0 mg) was ultrasonically dispersed in an 80% aqueous 2-propanol solution (2.0 mL) to form a homogeneous suspension. A portion of the dispersion was then cast on a GCE to form a homogeneous catalyst layer. Finally, the cast film on the electrode was air-dried under vacuum. Cyclic voltammetry (CV) measurements of the electrocatalysts were carried out at the scan rate of 50 mV s^−1^ in N_2_-saturated 0.1 M HClO_4_ solution. All the potentials were transformed to the reference hydrogen electrode (RHE).

### Gas diffusion electrode (GDE) fabrication

A GDE was prepared as follows. The electrocatalyst was dispersed in a 50 mL 2-propanol aqueous solution by sonication for 1 h, which was filtered using a carbon gas diffusion layer (GDL) as a filter paper. The obtained GDE was dried overnight under vacuum at 25 °C to remove the residual solvent.

### Preparation of PA doped-PBI membrane

200 mg of PBI was dissolved in DMAc (10 mL) by stirring for 1 day to completely dissolve the polymer. The resultant polymer solution was casted on a glass plate and gradually increased the temperature to 120 °C. The heating process of the formed film was further continued at 120 °C for another 5 h to assure the removal of the solvent and the PBI film was peeled off from the glass substrate. Finally, the membrane was doped in an 85-wt% phosphoric acid solution for 5 days. The membrane thickness of the obtained membrane was determined to be ~25 μm. The doping level calculated by the weight change of the dry membrane upon doping was 5 H_3_PO_4_ molecules/repeat unit of the PBI.

### Membrane electrode assembly (MEA) fabrication and FC testing

The MEA was prepared by hot pressing the GDE and the PBI membrane under 2 MPa at 120 °C for 30 s. The active area of the MEA was 1 cm^2^. The FC performance of the assembled MEA was evaluated at 120 °C without any external humidification using a Model 890e fuel cell test system. The polarization and the power density curves were measured under the atmospheric pressure by flowing dry hydrogen (flow rate; 100 mL min^−1^) and dry air (flow rate; 200 mL min^−1^) at the anode and the cathode, respectively.

### Durability test

The durability test was carried out by potential cycling from 1.0 V to 1.5 V *vs*. RHE in order to accelerate the carbon corrosion and Pd dissolution/aggregation. The anode and cathode were flowed by dry hydrogen and nitrogen during the potential cycling. After several cycles, the cathode was switched to air to measure the I-V curve.

## Additional Information

**How to cite this article**: Yang, Z. *et al.* High Performance Palladium Supported on Nanoporous Carbon under Anhydrous Condition. *Sci. Rep.* **6**, 36521; doi: 10.1038/srep36521 (2016).

**Publisher’s note:** Springer Nature remains neutral with regard to jurisdictional claims in published maps and institutional affiliations.

## Supplementary Material

Supplementary Information

## Figures and Tables

**Figure 1:**
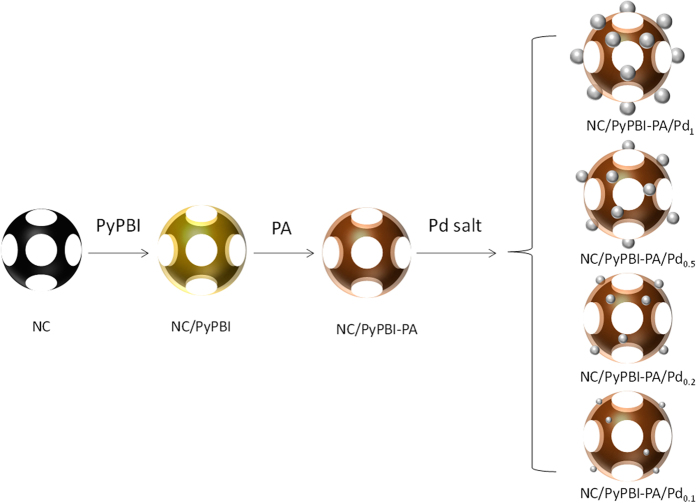
Procedure of preparation of the Pd electrocatalyst. Schematic illumination of the preparation of various NC/PyPBI-PA/Pd electrocatalysts.

**Table 1:** Comparison of power densities measured under high temperature using PA-PBI.

Anode (mg cm^−2^)	Cathode (mg cm^−2^)	Reactant	Temperature (°C)	Power Density (mW cm^−2^)	Ref.
0.05 (Pd)	0.45 (Pt)	H_2_/Air	120	188	This work
0.45 (Pt)	0.45 (Pt)	H_2_/Air	120	160	32
1 (Pt)	1 (Pt)	H_2_/Air	132	110	41
1.2 (Pt)	1.2 (Pt)	H_2_/Air	145	250	42
1.6 (Pt)	1.6 (Pt)	H_2_/Air	160	440	43
1 (Pt)	1 (Pt)	H_2_/Air	120	240	44
0.45 (Pt)	0.45 (Pt)	H_2_/Air	120	250	45

## References

[b1] Winter M. & Brodd R. J. What Are Batteries, Fuel Cells, and Supercapacitors? Chem. Rev. 104, 4245–4270 (2004). 15669155 10.1021/cr020730k

[b2] Borup R. *et al.* Scientific Aspects of Polymer Electrolyte Fuel Cell Durability and Degradation. Chem. Rev. 107, 3904–3951 (2007). 17850115 10.1021/cr050182l

[b3] Iiyama A. *et al.* K. FCEV development at Nissan. ECS Trans. 64, 11–17 (2014).

[b4] Stephens I. *et al.* Understanding the electrocatalysis of oxygen reduction on platinum and its alloys. Energy Environ. Sci. 5, 6744–6762 (2012).

[b5] Tasic G. S. *et al.* Non-noble metal catalyst for a future Pt free PEMFC. Electrochem. Commun. 11, 2097–2100 (2009).

[b6] Yang Z. *et al.* Design of Polymer-Coated Multi-Walled Carbon Nanotube/Carbon Black-based Fuel Cell Catalysts with High Durability and Performance Under Non-humidified Condition. Electrochim. Acta 170, 1–8 (2015).

[b7] Stephens I. E. L. *et al.* Understanding the electrocatalysis of oxygen reduction on platinum and its alloys. Energy Environ. Sci. 5, 6744–6762 (2012).

[b8] Wang Y.-J. *et al.* Carbon-Supported Pt-Based Alloy Electrocatalysts for the Oxygen Reduction Reaction in Polymer Electrolyte Membrane Fuel Cells: Particle Size, Shape, and Composition Manipulation and Their Impact to Activity. Chem. Rev. 115, 3433–3467 (2015). 25871490 10.1021/cr500519c

[b9] Yang Z. *et al.* NaOH-Aided Platinum Nanoparticle Size Regulation on Polybenzimidazole-Wrapped Carbon Nanotubes for Use as Non-Humidified Polymer Electrolyte Fuel Cell Catalyst. ChemCatChem 8, 268–275 (2016).

[b10] Veizaga N. S. *et al.* Development of PtGe and PtIn anodic catalysts supported on carbonaceous materials for DMFC. Int. J. Hydrogen Energy 39, 8728–8737 (2014).

[b11] Shimizu Y. *et al.* Effective Utilization of Carbon Nanocoil-supported PtRu Anode Catalyst by Applying Anode Microporous Layer for Improved Direct Methanol Fuel Cell Performance. Electrochemistry 83, 381–385 (2015).

[b12] Zhao L. *et al.* Facile one-pot synthesis of Pt/graphene-TiO2 hybrid catalyst with enhanced methanol electrooxidation performance. J. Power Sources 279, 210–217 (2015).

[b13] Wang J.-Y. *et al.* Boron-Doped Palladium Nanoparticles on Carbon Black as a Superior Catalyst for Formic Acid Electro-oxidation. J. Phys. Chem. C 113, 8366–8372 (2009).

[b14] Chang J. *et al.* Ni2P enhances the activity and durability of the Pt anode catalyst in direct methanol fuel cells. Energy Environ. Sci. 7, 1628–1632 (2014).

[b15] Holdcroft S. Fuel Cell Catalyst Layers: A Polymer Science Perspective. Chem. Mater. 26, 381–393 (2013).

[b16] Mauritz K. A. & Moore R. B. State of understanding of Nafion. Chem. Rev. 104, 4535–4585 (2004). 15669162 10.1021/cr0207123

[b17] Rodgers M. P. *et al.* Fuel cell perfluorinated sulfonic acid membrane degradation correlating accelerated stress testing and lifetime. Chem. Rev. 112, 6075–6103 (2012). 23061417 10.1021/cr200424d

[b18] Li Q. *et al.* J. Approaches and Recent Development of Polymer Electrolyte Membranes for Fuel Cells Operating above 100 °C. Chem. Mater. 15, 4896–4915 (2003).

[b19] Li Q. *et al.* High temperature proton exchange membranes based on polybenzimidazoles for fuel cells. Prog. Polym. Sci. 34, 449–477 (2009).

[b20] Asensio J. A., Sanchez E. M. & Gomez-Romero P. Proton-conducting membranes based on benzimidazole polymers for high-temperature PEM fuel cells. A chemical quest. Chem. Soc. Rev. 39, 3210–3239 (2010). 20577662 10.1039/b922650h

[b21] Yang Z., Moriguchi I. & Nakashima N. Durable Pt Electrocatalyst Supported on a 3D Nanoporous Carbon Shows High Performance in a High-Temperature Polymer Electrolyte Fuel Cell. ACS Appl. Mater. Interfaces 7, 9800–9806 (2015). 25902007 10.1021/acsami.5b01724

[b22] Fujigaya T., Okamoto M. & Nakashima N. Design of an assembly of pyridine-containing polybenzimidazole, carbon nanotubes and Pt nanoparticles for a fuel cell electrocatalyst with a high electrochemically active surface area. Carbon 47, 3227–3232 (2009).

[b23] Okamoto M., Fujigaya T. & Nakashima N. Design of an assembly of poly(benzimidazole), carbon nanotubes, and Pt nanoparticles for a fuel-cell electrocatalyst with an ideal interfacial nanostructure. Small 5, 735–740 (2009). 19263429 10.1002/smll.200801742

[b24] Yang Z., Berber M. R. & Nakashima N. A polymer-coated carbon black-based fuel cell electrocatalyst with high CO-tolerance and durability in direct methanol oxidation. J. Mater. Chem. A 2, 18875–18880 (2014).

[b25] Yang Z. *et al.* Facile Enhancement in CO-Tolerance of a Polymer-Coated Pt Electrocatalyst Supported on Carbon Black: Comparison between Vulcan and Ketjenblack. ACS Appl. Mater. Interfaces 7, 15885–15891 (2015). 26147674 10.1021/acsami.5b03371

[b26] Yang Z. *et al.* An Enhanced Anode based on Polymer-Coated Carbon Black for use as a Direct Methanol Fuel Cell Electrocatalyst. ChemCatChem 7, 808–813 (2015).

[b27] Yue H. *et al.* Ethylene glycol: properties, synthesis, and applications. Chem. Soc. Rev. 41, 4218–4244 (2012). 22488259 10.1039/c2cs15359a

[b28] Fujigaya T. *et al.* Palladium-based anion-exchange membrane fuel cell using koh-doped polybenzimidazole as the electrolyte. ChemPlusChem 79, 400–405 (2014). 10.1002/cplu.201300377 31986600

[b29] Lee M. *et al.* Durability of Pt/graphitized carbon catalyst prepared by the nanocapsule method for the start/stop operating condition of polymer electrolyte fuel cells. Electrochemistry 79, 381–387 (2011).

[b30] Hara M. *et al.* Electrochemical and Raman spectroscopic evaluation of Pt/graphitized carbon black catalyst durability for the start/stop operating condition of polymer electrolyte fuel cells. Electrochim. Acta 70, 171–181 (2012).

[b31] Maiyalagan T. Silicotungstic acid stabilized Pt–Ru nanoparticles supported on carbon nanofibers electrodes for methanol oxidation. Int. J. Hydrogen Energy 34, 2874–2879 (2009).

[b32] Gyenge E., Atwan M. & Northwood D. Electrocatalysis of Borohydride Oxidation on Colloidal Pt and Pt-Alloys (Pt-Ir, Pt-Ni, and Pt-Au) and Application for Direct Borohydride Fuel Cell Anodes. J. Electrochem. Soc. 153, A150–A158 (2006).

[b33] Selvaraj V. & Alagar M. Pt and Pt–Ru nanoparticles decorated polypyrrole/multiwalled carbon nanotubes and their catalytic activity towards methanol oxidation. Electrochem. Commun. 9, 1145–1153 (2007).

[b34] Yang Z. & Nakashima N. An Electrocatalyst Based on Carbon Nanotubes Coated with Poly(vinylpyrrolidone) Shows a High Tolerance to Carbon Monoxide in a Direct Methanol Fuel Cell. ChemCatChem 8, 600–606 (2016).

[b35] Yang Z., Fujigaya T. & Nakashima N. A phosphoric acid-doped electrocatalyst supported on poly(para-pyridine benzimidazole)-wrapped carbon nanotubes shows a high durability and performance. J. Mater. Chem. A 3, 14318–14324 (2015).

[b36] Mougenot M. *et al.* PdAu/C catalysts prepared by plasma sputtering for the electro-oxidation of glycerol. Appl. Catal. B 107, 372–379 (2011).

[b37] Zhang J. *et al.* Boosting Electrocatalytic Performances of Palladium Nanoparticles by Coupling with Metallic Single-Walled Carbon Nanotubes. Chem. Mater. 26, 2789–2794 (2014).

[b38] Yu S. & Benicewicz B. C. Synthesis and properties of functionalized polybenzimidazoles for high-temperature PEMFCs. Macromolecules 42, 8640–8648 (2009).

[b39] Moriguchi I. *et al.* Colloidal crystal-templated porous carbon as a high performance electrical double-layer capacitor material. Electrochem. Solid-State Lett. 7, A221–A223 (2004).

[b40] Xiao L. *et al.* Synthesis and characterization of pyridine-based polybenzimidazoles for high temperature polymer electrolyte membrane fuel cell applicationsx. Fuel Cells 5, 287–295 (2005).

[b41] Tang Q. *et al.* Phosphoric acid-imbibed three-dimensional polyacrylamide/poly(vinyl alcohol) hydrogel as a new class of high-temperature proton exchange membrane. J. Power Sources 229, 36–41 (2013).

[b42] Wannek C., Lehnert W. & Mergel J. Membrane electrode assemblies for high-temperature polymer electrolyte fuel cells based on poly(2,5-benzimidazole) membranes with phosphoric acid impregnation via the catalyst layers. J. Power Sources 192, 258–266 (2009).

[b43] Guo L. *et al.* Embedding Pt Nanocrystals in N-Doped Porous Carbon/Carbon Nanotubes toward Highly Stable Electrocatalysts for the Oxygen Reduction Reaction. ACS Catal. 5, 2903–2909 (2015).

[b44] Li X., Chen X. & Benicewicz B. C. Synthesis and properties of phenylindane-containing polybenzimidazole (PBI) for high-temperature polymer electrolyte membrane fuel cells (PEMFCs). J. Power Sources 243, 796–804 (2013).

[b45] Berber M. R. *et al.* Remarkably Durable High Temperature Polymer Electrolyte Fuel Cell Based on Poly(vinylphosphonic acid)-doped Polybenzimidazole. Sci. Rep. 3, 1736 (2013).

