# Supplementary material for: High Performance Palladium Supported on Nanoporous Carbon under Anhydrous Condition
Source: Sci Rep. 2016 Nov 4;6:36521. doi: 10.1038/srep36521 (PMC5109910; doi:10.1038/srep36521)
Supplement: Supplementary Information [file srep36521-s1.pdf]

Supplementary Information

High Performance Palladium Supported on Nanoporous Carbon under Anhydrous Condition

Zehui Yang[a], Ying Ling[a,] Yunfeng Zhang[a*] and Guodong Xu[a*]

[a]*Sustainable Energy Laboratory, Faculty of Materials Science and Chemistry, China University of Geosciences Wuhan, 388 Lumo RD, Wuhan, 430074, China*

To whom correspondence should be addressed:

E-mail: zhangyf329@gmail.com

xuguodong003@gmail.com

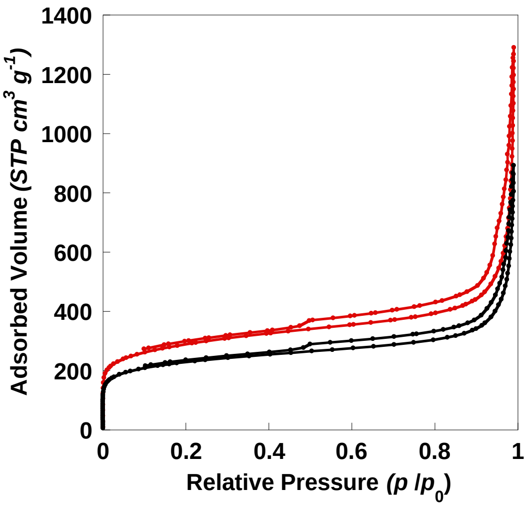

Figure S1| N_2 adsorption/desorption isotherms of the NanoPC (red line) and NanoPC/PyPBI (black line).

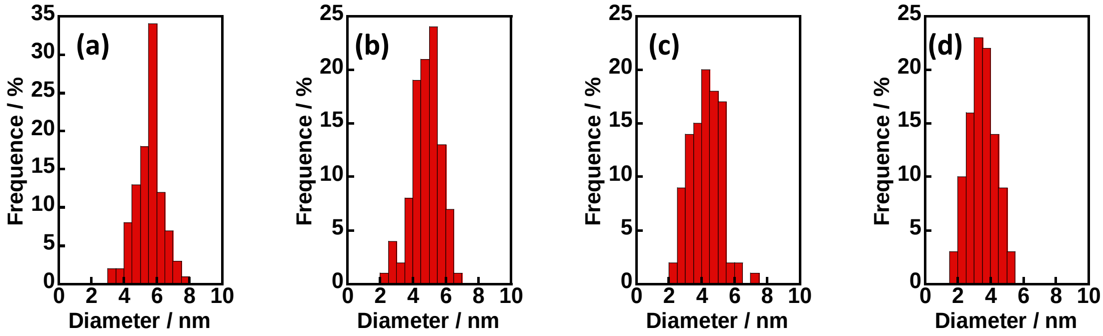

Figure S2| Histograms of Pd-NP size distribution of NC/PyPBI-PA/Pd_1 (a), NC/PyPBI-PA/$Pd_{0.5}$ (b), NC/PyPBI-PA/$Pd_{0.2}$ (c) and NC/PyPBI-PA/$Pd_{0.1}$ (d), respectively.

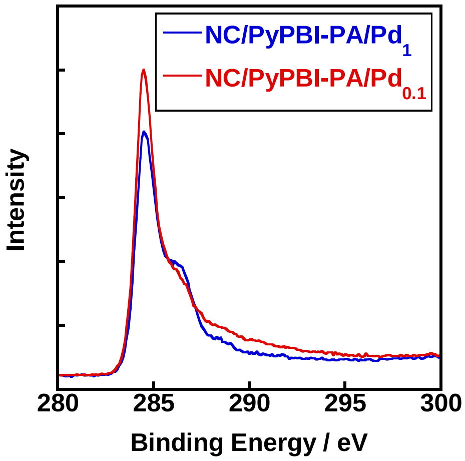

Figure S3| XPS narrow scan of NC/PyPBI-PA/Pd_1 and NC/PyPBI-PA/$Pd_{0.1}$ in the C_{1s} region.

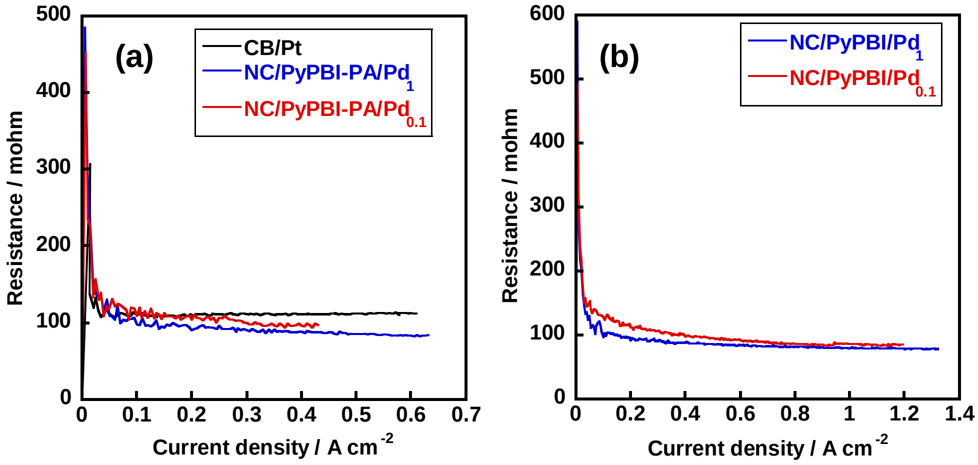

Figure S4| (a) Resistances of the MEAs fabricated from commercial CB/Pt, NC/PyPBI-PA/Pd_1 and NC/PyPBI-PA/$Pd_{0.1}$, respectively. (b) Resistances of hybrid MEAs fabricated from NC/PyPBI-PA/Pd_1 and NC/PyPBI-PA/$Pd_{0.1}$ as anode and commercial CB/Pt as cathode.

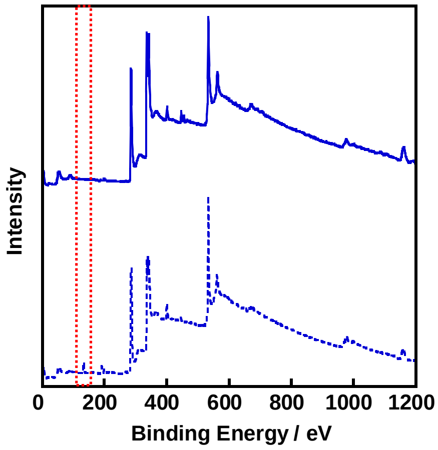

Figure S5| XPS survey scans of NC/PyPBI/Pd$_1$ (solid line) and NC/PyPBI-PA/Pd$_1$ (dotted line), respectively.

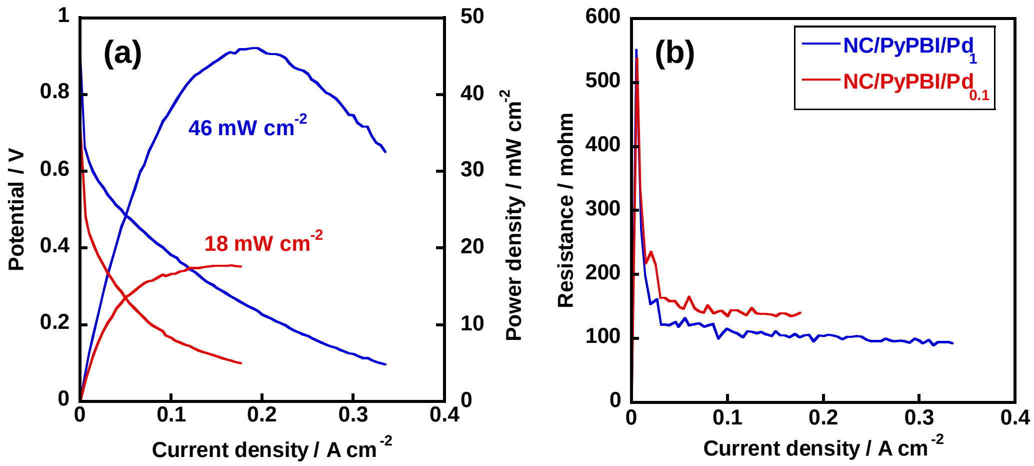

Figure S6| I-V, power density (a) and resistances (b) of the MEAs fabricated from NC/PyPBI/Pd$_1$ and NC/PyPBI/Pd$_{0.1}$, respectively.

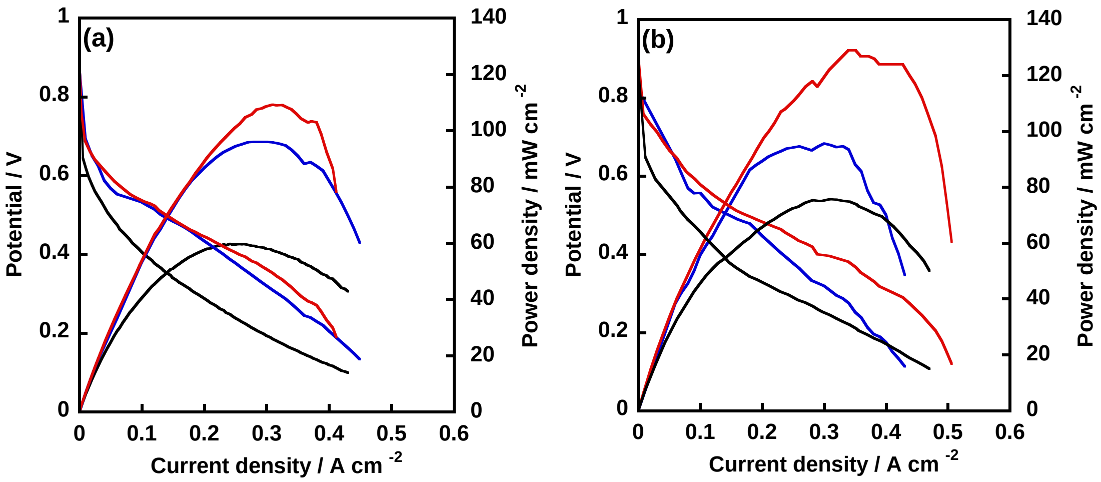

Figure S7| I-V and power density curves of the MEAs fabricated from NC/PyPBI-PA/$Pd_{0.1}$ and NC/PyPBI-PA/$Pd_{0.1}$/PVPA at 120 ^{o}C (black line), 140 ^{o}C (blue line) and 160 ^{o}C (red line), respectively.

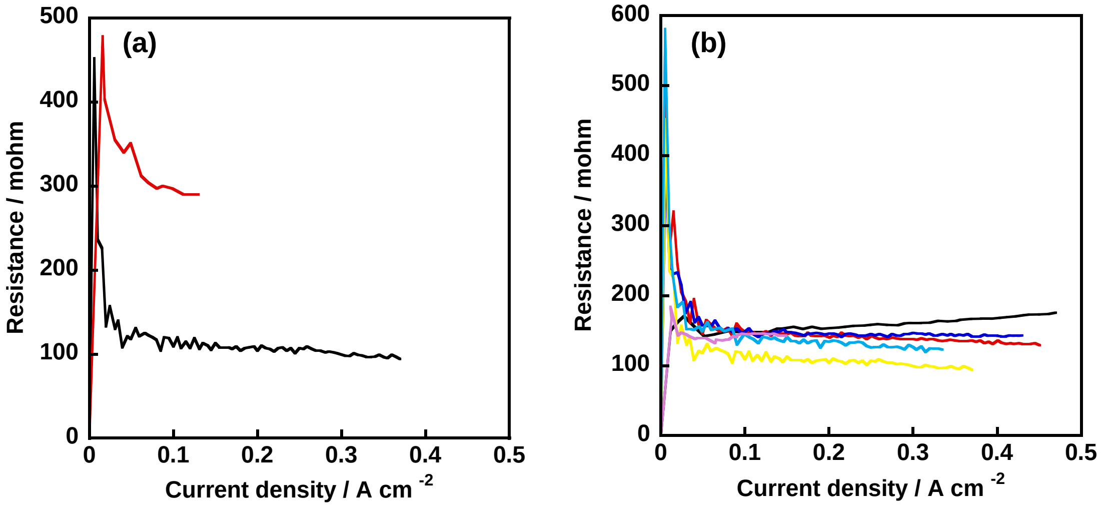

Figure S8| Resistances of NC/PyPBI-PA/$Pd_{0.1}$ (a) and NC/PyPBI-PA/$Pd_{0.1}$/PVPA (b) during the durability test.
